# *In vitro* performance of Lifetech IBS Angel™ (iron-based bioresorbable scaffold) stents during overdilation for use in pediatric patients

**DOI:** 10.3389/fcvm.2022.1006063

**Published:** 2022-11-09

**Authors:** Kurt Bjorkman, Jennifer R. Maldonado, Stephanie Saey, Daniel McLennan

**Affiliations:** ^1^Roy J. and Lucille A. Carver College of Medicine, Iowa City, IA, United States; ^2^Medical College of Wisconsin, Milwaukee, WI, United States

**Keywords:** bioresorbable scaffolds (BRS), stress testing device, foreshortening, stent, congenital heart disease

## Abstract

**Objectives:**

The objective of this study was to assess the mechanical performance of the Lifetech IBS Angel stents during overdilation as is often required in pediatric applications; including time of first fracture, foreshortening, and the type of fracturing that occurs.

**Materials and methods:**

*In vitro* testing was performed and repeated for each stent three times under physiologic conditions with continuous audiovisual imaging allowing for post-testing evaluations. Assessment of sheath fit was also completed.

**Results:**

A total of 47 stents on monorail system were overdilated to complete fracture after passing through either a 4 or 5 French sheath. First strut fracture occurred in 4 and 6 mm stents when they reached greater than 50% overexpansion. Larger stents could achieve at least 30% increased diameter prior to first strut fracture. No fragmentation of any of the stents was seen throughout testing.

**Conclusion:**

The IBS Angel has thin struts allowing for a lower profile with increased maneuverability and use with smaller sheaths. Embolization potential of strut fragments was not seen. Increased diameter well beyond design parameters was seen in all with acceptable foreshortening.

## Introduction

Stent angioplasty in pediatric populations carries some unique challenges that are not seen in adult populations. In addition to the technical difficulty of performing procedures in physically smaller patients, pediatric interventionalists must also consider the need to keep up with future somatic vessel growth after stent deployment and the chance that the stent may need to be removed when used as temporary palliation before surgery for congenital cardiac defects (1). Bioresorbable stents offer a potential as a temporary palliation while also having a predictably limited lifespan that may lessen some of the challenges of future procedures that can be seen with permanent stents (2, 3).

**FIGURE 1:**
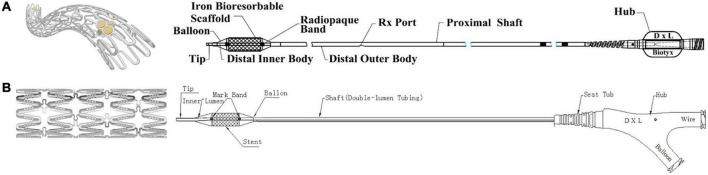
Lifetech iron-based bioresorbable scaffold stent and angioplasty catheter. **(A)** Delivery system for stent diameters 2.25–4.0 mm. **(B)** Delivery system for stent diameters 5.0–10.0 mm.

The ideal bioresorbable stent has been proposed to have thin struts, a low profile for maneuverability and use with smaller sheaths, while maintaining adequate radial strength to maintain vessel patency after deployment (4–8). These stents should also ideally have no or low thrombogenic risks and demonstrate complete absorption within a predicted timeframe. The Lifetech IBS (Iron-based Bioresorbable Scaffold) Angel stent [Biotyx Medical (Shenzhen, China) Co. Ltd (a company of Lifetech Scientific)] (Figure 1) is made specifically for pediatric applications from nitrided iron through vacuum plasma nitriding to produce a thin, low-profile stent with a thickness of 70 μm. The nitriding process additionally increases its mechanical strength, radial strength, stiffness, and microhardness comparable to previous iron-based stents, and controls the microstructure to increase its *in vitro* corrosion rate (9, 10).

Previous *in vivo* testing (9, 10) has demonstrated vessel patency 3 and 6 months after placement with slight luminal loss from intimal hyperplasia, and relative stenosis of vessels in piglets at 12 months with no thrombosis or local tissue necrosis. Similar *in vivo* testing in rabbit abdominal aortas at 1, 6, and 12 months has shown slight inflammatory response with no tissue necrosis of neointima or its underlying layers by histopathological observation, and macrophages can be seen engulfing corrosion particles around the strut at 12 months. *In vivo* corrosion profiles of this nitrided stent have demonstrated mass loss of 44.5 ± 6.4% at 12 months and 62.9 ± 9.0% at 24 months which is faster than pure iron-based stents, while also having higher radial strength at 6 and 9 months—above 150 kPa and above 120 kPa, respectively.

As the Lifetech IBS Angel™ stent with its low-profile design has demonstrated favorable strength and corrosion profiles in both *in vitro* and *in vivo* testing with limited-to-no thrombogenic risk, there is promise that it may provide a reasonable bioresorbable stent option (9–11). Prior to in-human use in pediatrics, this work seeks to explore how these stents will perform with overdilation as is often needed in pediatric patients to keep up with somatic growth. The aim of this study is to define the point of overdilation when first strut fracture can be expected, and to quantify the degree of foreshortening seen with overdilation prior to fracture with secondary aims to evaluate how these stents fracture, their risk of embolization, and sheath fit.

## Materials and methods

### Stent dilation and overdilation

**FIGURE 2:**
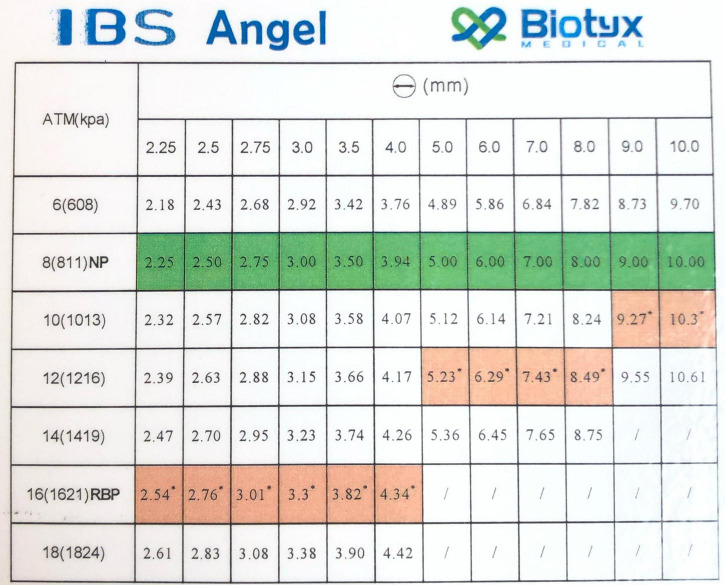
IBS Angel monorail stent diameter to balloon pressure ratio.

**FIGURE 3:**
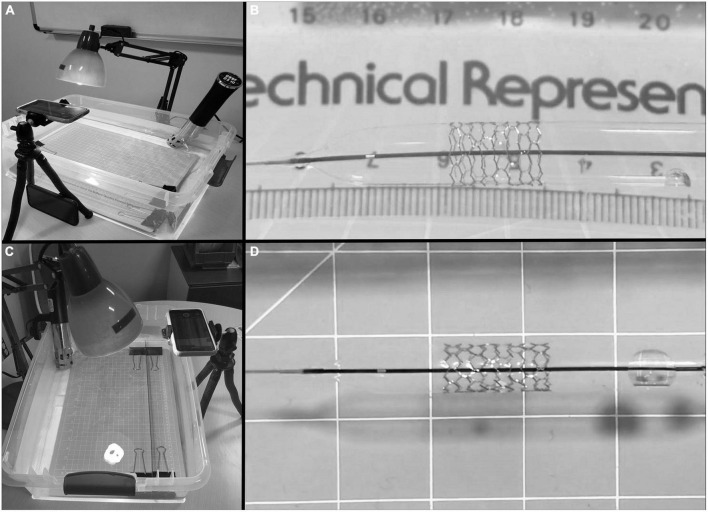
Water bath set up with immersion circulator and camera stand. **(A)** Lateral view of test setup with water bath, immersion circulator, lighting, and camera stand. **(B)** Lateral view of stent during dilation. **(C)** Overhead view of test setup. **(D)** Anterior-posterior view of stent during dilation.

Premounted monorail iron-based bioresorbable stents {Lifetech IBS (Iron-based Bioresorbable Scaffold) Angel™ stent [Biotyx Medical (Shenzhen, China) Co. Ltd (a company of Lifetech Scientific)]} were obtained from the manufacturer in various diameters and lengths (Table 1) along with (Figure 2) stent diameter to balloon pressure ratio. All stent dilations and serial overdilation were completed in a water bath made from a clear 60.3 cm × 40.6 cm × 17.5 cm container (Sterilite, USA), filled with approximately 20 L of water heated with an immersion circulator (InstaPot Model: Accu SSV800, USA) to 37°C to mimic physiologic conditions and similar to previously published protocols (12, 13; Figure 3). All dilations were recorded from superior and lateral views with continuous audiovisual recordings in 1080p resolution at 60 fps. Stents were immersed in water bath and then expanded on premounted balloons and serially over dilated on angioplasty balloons as shown in Table 2. During dilations, each increase in balloon pressure by 2 atm was noted allowing for post-test analysis at sequentially increasing pressures starting 2 atm below nominal pressure and going to 2 atm above rated burst pressure for each balloon. If there was no stent fracture with dilation, the next larger balloon was then used for further overdilation. The point of first strut fracture was noted and then further dilation completed for qualitative assessment of complete fracture of all struts. Strut facture was clearly noticeable by an audible and tactile pop that was confirmed by continuous high-definition imaging from superior and lateral views at 60fps. The point of first strut fracture and complete facture of all struts were confirmed by manual inspection and with analysis of the audiovisual recordings. Each angioplasty catheter was separately imaged next to a calibrated ruler to allow for calibration of measurement software during analysis.

**TABLE 1:** Number of each stent size tested.

	Stent diameter*			
	
Stent length	8 mm	12 mm	15 mm	38 mm
4 mm (4 mm)	3	3	3	3
6 mm (5 mm)	3	3	3	3
8 mm (7 mm)	3	3	3	3
10 mm (9 mm)	3	2	3	3

*Diameter in parentheses denotes diameter of premounted monorail angioplasty catheter.

**TABLE 2:** Balloons used for dilation and overdilating stents.

	Balloon diameter (mm)								
	
Stent diameter (mm)	4	5	6	7	8	9	10	12	14
4	X		X						
6		X	X	X	X		X		
8				X	X		X	X	
10						X	X	X	X

X denotes use of balloon for stent dilation.

### Sheath fit

Prior to dilation, each premounted monorail stent was passed through a short sheath to test for ease of fit and then removed through the sheath after deployment of the stent with qualitative assessment of resistance (minimal, some, difficult, not possible). The 4 mm and 6 mm diameter stents were tested through a 4-French Prelude Ideal hydrophilic sheath (Merrit Medical, UT, USA), and the 8 and 10 mm stents tested through a 5-French Prelude Ideal hydrophilic sheath (Merrit Medical, UT, USA).

### Post-test measurements and analysis

Post-test video analysis was completed by creating still frame images of stents and balloons at each 2 atm increment throughout inflation with video editing software in 1080p resolution (Filmora Pro, Wondershare Technology Group Co., Ltd). All measurements were then completed to the 0.01 mm on still images from superior and lateral views using ImageJ software (U.S National Institutes of Health, Bethesda, MD, USA). The angioplasty catheter tips were first measured and then used to calibrate stent measurements throughout analysis.

At each 2 atm pressure increment, stent length and diameter were measured from superior and lateral view, and then means calculated for each stent size throughout dilation and overdilation. Stent dimensions were measured at point of first fracture, with mean and 95% confidence interval (CI) calculated for each stent size. Mean percent foreshortening was calculated for each stent size throughout dilation [(stent length − reported length)/reported length]. Mean percent foreshortening for each stent size was then also plotted against stent diameter throughout dilation.

## Results

**FIGURE 4:**
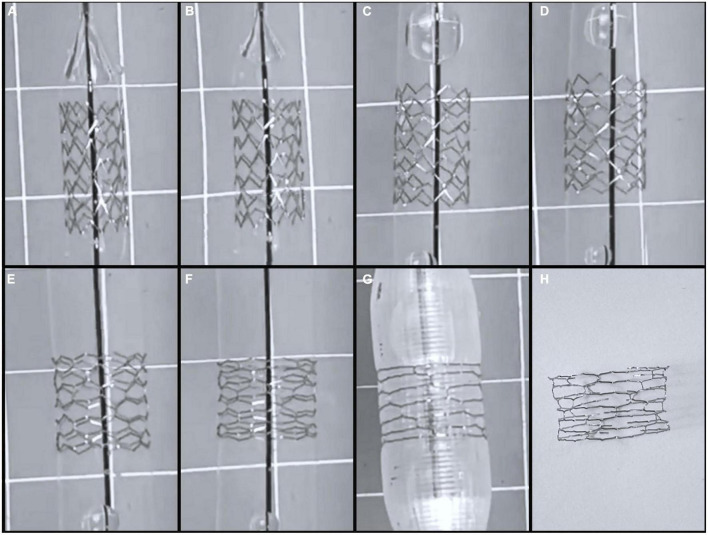
Serial dilation of 8 mm by 15 mm Lifetech Iron-Based Bioresorbable Scaffold Angel stent. **(A)** 8 mm stent on premounted 7 mm balloon at 6 atm, **(B)** premounted 7 mm balloon at 12 atm, **(C)** 8 mm balloon at 6 atm, **(D)** 8 mm balloon at 12 atm, **(E)** 10 mm balloon at 6 atm, **(F)** 10 mm balloon at 12 atm, **(G)** 12 mm balloon at 6 atm which was point of first strut fracture. **(H)** Stent post complete strut fracture.

**FIGURE 5:**
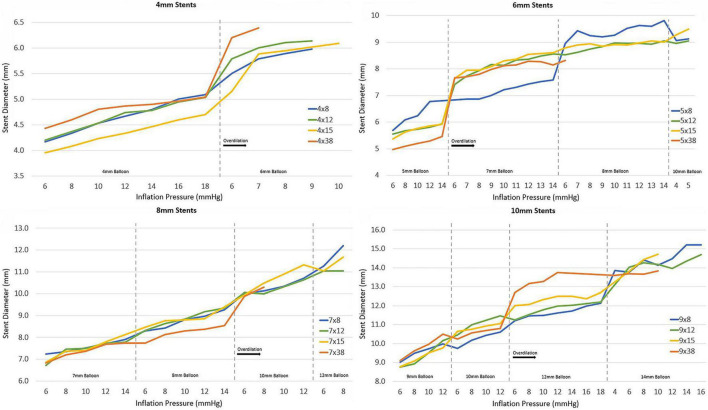
Mean stent diameter with dilation and overdilation until first strut fracture.

**FIGURE 6:**
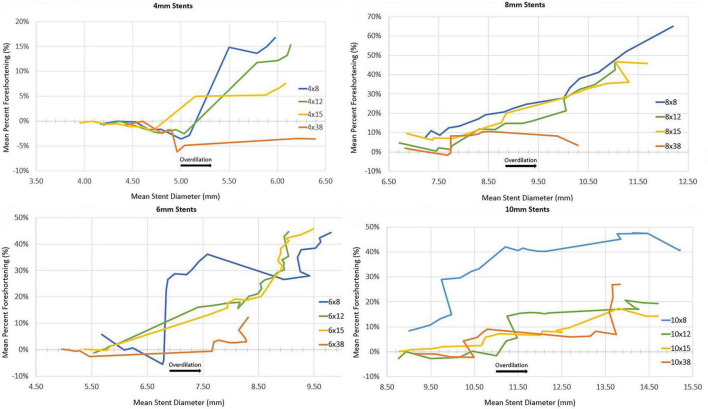
Mean percent foreshortening with increasing mean stent diameter.

A total of 47 stents were tested (Table 1). Mean stent diameter for each stent size throughout dilation and overdilation until first strut fracture shown in Figures 4, 5. The point of first strut fracture for each strut fracture with 95% CI for each stent size shown in Table 3. After first strut fracture, complete fracture of all struts was completed easily with further dilations. Mean foreshortening percent (%) at time of first fracture is shown in Table 3 and mean foreshortening % is shown plotted against mean stent diameters in Figure 6 for each stent size.

**TABLE 3:** Percent increase in stent diameter and percent foreshortening at time of first strut fracture.

	Diameter % increase at time of fracture		Foreshortening % at time of fracture	
		
Stent size	% Increase	95% CI	% Foreshortened	95% CI
4 × 8	49%	46–53%	28%	22–35%
4 × 12	57%	59–58%	27%	13–42%
4 × 15	54%	48–59%	20%	17–24%
4 × 38	60%	57–63%	0%	0%
6 × 8	53%	49–58%	48%	42–54%
6 × 12	51%	49–53%	52%	39–65%
6 × 15	60%	42–78%	46%	39–52%
6 × 38	40%	37–43%	14%	5–23%
8 × 8	42%	30–54%	59%	44–74%
8 × 12	39%	36–43%	46%	41–51%
8 × 15	45%	41–49%	47%	44–50%
8 × 38	24%	15–34%	8%	0–21%
10 × 8	45%	37–52%	48%	40–56%
10 × 12	47%	39–55%	19%	19–20%
10 × 15	33%	18–48%	15%	7–23%
10 × 38	37%	35–40%	26%	24–27%

Qualitative assessment of complete strut fracture was performed for all stents and demonstrated easy fracture of all strut rings after initial strut fracture. There were no small stent fragments produced by complete stent fracture. However, two of the 6 × 38 mm stents fractured into two separate large stent segments with complete fracture, and all three of the 8 × 38 mm stents fractured into large segments with complete fracture of all struts (2 stents into 3 large pieces and the third stent of this size into 2 large pieces). All other stents, including the 4 × 38 mm, the 10 × 38 mm stents, remained in one large piece with complete strut fracture.

There was one balloon rupture that occurred with one of the 6 × 8 mm angioplasty catheters at 12 atm, which is the rated burst pressure for this balloon. All other balloons tested went to 2 atm above burst pressure without rupture or leak.

During sheath fit testing of the monorail system, all 4 and 6mm diameter stents fit easily through a 4-French Prelude ideal sheath and balloons were removed through sheaths after stent deployment with minimal or no resistance. All 8 and 10 mm diameter stents fit easily through a 5-French prelude ideal sheath and balloons were removed through sheaths after stent deployment with minimal or no resistance.

## Discussion

Throughout *in vitro* testing of the Lifetech IBS Angel™ monorail stents in this study, they were found to deploy in a predictable fashion, performing well throughout the full range of anticipated diameters for use without significant foreshortening or strut fracture until well beyond design parameters for these stents. Stent dilation and overdilation demonstrated promising functionality when considering their potential use in pediatric patients. Specifically, almost all the 4mm and 6mm stents reached greater than 50% of their designed diameter, and all stents tested except for the 8 × 39 mm stents reached diameters greater than 30% of their designed diameter prior to first strut fracture.

Regarding foreshortening seen with overdilation, the stents had what was deemed to be an acceptable amount of foreshortening, with a foreshortening % less than 20% when the diameter was increased to 20% greater than designed diameter seen in all stent sizes except for the 5 × 8 mm stents. In general, the shorter length stents had greater foreshortening than the longer stents, while the longer stents had first fracture at lesser degrees of overdilation.

When considering potential embolism during stent fracture with overdilation, there were no small stent fragments produced with complete strut fracture of any stent. It was noted, however, that many of the stents with lengths of 38 mm did break into 2 or 3 large segments with complete fracture (Table 4).

**TABLE 4:** Diameter of angioplasty catheter used for complete fracture of all struts by stent size.

Stent diameter	Largest balloon used without complete strut fracture±	Balloon size used to achieve complete strut fracture
4 mm	6 mm	7 mm
6 mm	8 mm*	10 mm
8 mm	10 mm	12 mm
10 mm	14 mm	16 mm

± Uniform performance of stents in each diameter size was seen unless otherwise noted.

*1 of 36 × 38 mm stents had complete strut fracture with 8 mm balloon, all other 6 mm stents required 10 mm balloon for complete fracture.

This study provides data to support the safety and utility of these devices with overdilation. Its strength includes the wide variety of stent diameters and lengths tested, including an in-depth qualitative analysis with high-definition video of how these stents fracture when expanded to terminal diameters. There are multiple congenital or progressive anatomic pathologies that might be well-suited for the use of bioresorbable stents. Stent angioplasty of a narrow right-ventricular outflow tract is frequently used to avoid neonatal repair in Tetralogy of Fallot with inadequate pulmonary blood flow, but then presents the additional challenge of surgical stent resection at the time of complete surgical repair (14). Stenting of the ductus arteriosus is an additional palliative transcatheter procedure performed in congenital defects with limited pulmonary blood flow such as pulmonary atresia, or in conditions that require additional blood flow from the right ventricle to support systemic circulation such as in hypoplastic left heart syndrome (15–18). In both of these conditions, the placement of a metal stent can require extensive tissue resection and increased surgical augmentation of pulmonary arteries at time of surgery. The use of a bioresorbable stent in these applications could potential offer initial non-invasive palliation similar to their non-bioresorbable counterparts, while potentially limiting the surgical challenges that metal stents can create. Pulmonary vein stenosis is an additional condition addressed in pediatric cardiac catheterization labs that requires numerous repeat interventions when multiple-vessel disease is present (19, 20). In these cases, there is improved outcomes when vein diameters of 7 mm or great or reached. However, smaller stents are often needed to be used initially and then fractured to allow for larger stents at a later date. The use of bioresorbable stents could potentially lessen the challenges of needing to fracture old stents when placing larger ones at a later date. Ideally, the placement of a bioresorbable stent might allow for stable vessel patency, allowing for somatic growth of the child and then placement of a large stent at a later date.

Limitations of this study include the *in vitro* nature of the tests. There may be some degree of stent stabilization that occurs when explaining stents while they are against the luminal wall of a vessel, or conversely, non-uniform pressure against the exterior of a stent may cause it to fracture at a point earlier than was seen in these *in vitro* tests. The next steps for evaluation of these stents are further in human use. We have begun use of these stents in small children with pulmonary vein stenosis and right ventricular outflow tract obstruction.

Overall, the Lifetech IBS Angel stent appears to provide a potential bioresorbable stent option for angioplasty in pediatric patients with favorable performance throughout its designed diameters and the ability to be over dilate beyond these parameters with an acceptable degree of foreshortening.

## Data availability statement

The raw data supporting the conclusions of this article will be made available by the authors, without undue reservation.

## Author contributions

All authors listed have made a substantial, direct, and intellectual contribution to the work, and approved it for publication.
