# Supplementary material for: In vitro performance of Lifetech IBS Angel™ (iron-based bioresorbable scaffold) stents during overdilation for use in pediatric patients
Source: Front Cardiovasc Med. 2022 Nov 9;9:1006063. doi: 10.3389/fcvm.2022.1006063 (PMC9682241; doi:10.3389/fcvm.2022.1006063)
Supplement: Supplementary file 1 [file Data_Sheet_1.DOCX]

Protocol for In-Vitro Serial Balloon Dilation of LifeTech IBS Angel Stent

1. Prepare water bath by filling a clear 60.3cm x 40.6cm x 17.5cm container (Sterilite, USA) with approximately 20 liters of tap water.
2. Heat water bath with an immersion circulator (InstaPot Model: Accu SSV800, USA) to 37° Celsius to mimic physiologic conditions.
3. Continuous video monitoring in place with stands as shown in Figure 2. Audio and visual recordings obtained in 1080p resolution at 60fps.
4. Removing *premouted monorail* stent from company packaging, insert through sheath over monorail system and into heated water bath, with angioplasty balloon/stent in place for visualization throughout dilation and over-dilation. Qualitative assessment of ease of fit through sheath is noted at this time.
5. After attaching insufflator, inflate angioplasty balloon pausing at each increase of 2 atm, and continuing to inflate until 2atm above burst pressure as reported on manufacturer provided IFU. (FigureVI)
6. With completion of inflation, angioplasty balloon is deflated and removed from water bath, leaving stent in bath, and removing balloon through sheath. Qualitative assessment of ease of balloon removal through sheath is noted at this time.
7. Next increasing diameter of angioplasty catheter is then selected and placed in the water bath with previously expanded stent centered over angioplasty balloon.
8. Steps 5, 6, and 7 are then repeated with continuous observation for audible and tactile pop of first strut fracture, and then also until complete fracture of all stent struts.
9. Stents are manually inspected and imaged after initial fracture.
10. After complete fracture, stents are manually inspected and imaged again confirming complete fracture of all struts.
11. Visual inspection of water basin for any stent fragments after complete stent fracture
12. Post-test frame-by-frame analysis of high-definition video is then completed, confirming point of first strut fracture.
13. ImageJ software is then utilized to measure stent diameter and length at each 2atm of balloon inflation throughout testing, and also at point of first fracture and point of complete fracture. All measurements calibrated off of ruler present in video recordings and also off of known catheter tip length.
